# Complete Genome Sequences of Two Newcastle Disease Virus Strains Isolated from a Wild Duck and a Pigeon in Russia

**DOI:** 10.1128/genomeA.01348-16

**Published:** 2016-12-08

**Authors:** Marsel R. Kabilov, Tatyana Y. Alikina, Kseniya S. Yurchenko, Alexandra V. Glushchenko, Konstantin V. Gunbin, Alexander M. Shestopalov, Natalya V. Gubanova

**Affiliations:** a SB RAS Genomics Core Facility, Institute of Chemical Biology and Fundamental Medicine, Siberian Branch of the Russian Academy of Sciences, Novosibirsk, Russia; b FSBSI Research Institute of Experimental and Clinical Medicine, Novosibirsk, Russia; c Institute of Cytology and Genetics, Siberian Branch of the Russian Academy of Sciences, Novosibirsk, Russia; d Novosibirsk State University, Novosibirsk, Russia

## Abstract

Here, we report the complete genome sequences of two Newcastle disease virus (NDV) isolates, Adygea/duck/12/2008, from a wild duck in Russia, and Altai/pigeon/777/2010, from a pigeon in Russia. Based on comparative sequence analysis of the F gene, these strains were classified as NDV class II, genotypes VIId and VIb/2, respectively.

## GENOME ANNOUNCEMENT

Newcastle disease is a highly contagious viral disease of birds caused by virulent avian paramyxovirus serotype 1 (APMV-1), also known as Newcastle disease virus (NDV) (1). Antigenic variants of NDV have also appeared in pigeons (2) and wild duck (3).

In the present study, we report the complete genome sequences of two NDVs: Adygea/duck/12/2008, isolated from a wild duck in the Southern Federal District (Adygea) of Russia in 2008, and Altai/pigeon/777/2010, isolated from a rock dove (*Columba livia*) from Southern Siberia (Altai Republic) in Russia in 2010.

The NDV strains were propagated in the allantoic cavities of nine-day-old embryonated hen eggs obtained from nonvaccinated chickens. NDVs have been categorized into velogenic (highly virulent), mesogenic (intermediately virulent), and lentogenic (nonvirulent) pathotypes according to standard pathogenicity tests (1, 4). Strain Adygea/duck/12/2008 was classified as a velogenic NDV with a mean death time (MDT) of 50 h and with an intracerebral pathogenicity index (ICPI) of 1.55. Strain Altai/pigeon/777/2010 belongs to the mesogenic pathotype with an MDT of 75 h and an ICPI of 0.61.

RNAs were isolated from 100 to 400 µL of chicken allantois fluid with cultured viral particles using a GeneJET viral DNA/RNA purification kit (Thermo Fisher Scientific) and were treated with TURBO DNase (Thermo Fisher Scientific). Up to 40 ng of RNA were used for the DNA library, which was prepared using a TruSeq RNA sample preparation kit version 2 (Illumina). The DNA libraries were sequenced using the MiSeq platform (2 × 300-bp cycles; Illumina). The full-length genomes were assembled *de novo* with CLC Genomics Workbench version 8.5 (Qiagen). The complete genomes of NDV Adygea/duck/12/2008 and Altai/pigeon/777/2010 have been found to be 15,190 and 15,192 nucleotides in length, respectively.

The comparative analysis of the F protein-coding gene revealed that both NDV Altai/pigeon/777/2010 and Adygea/duck/12/2008 strains belong to NDV class II (4) and contain a polybasic amino acid sequence (^112^KRQKRF^117^ and ^112^RRQKRF^117^, respectively) at the F protein cleavage site. It was shown that the F gene from Altai/pigeon/777/2010 is similar to NDV isolate Pi/Rus/Kemerovo/0267/09 (98% sequence similarity), which belongs to the VIb/2 genotype (5). Genotype VI includes viruses that have been isolated from multiple avian species (6–10). Viruses of this genotype are particularly important because of their frequent association with doves and pigeons and the consequent risk for introduction into poultry flocks (6, 11, 12, 13).

The F gene of strain Adygea/duck/12/2008 is homologous to NDV strain Muscovy GI 227460816 (97.47% sequence similarity) (4), which belongs to the VIId genotype of NDV class II. Currently, genotype VII is the genotype most frequently associated with outbreaks of Newcastle disease in the Middle East and Asia (14), and outbreaks caused by these viruses are of particular concern given that some strains have shown increased virulence in poultry, while others have expanded their host range and are now able to cause disease in geese (9, 15). Additionally, an outbreak of Newcastle disease in South America has been recently attributed to a genotype VII virus, indicating that these viruses are spreading to other locations around the world (16).

### Accession number(s).

The complete genome sequences of NDV Adygea/duck/12/2008 and Altai/pigeon/777/2010 have been deposited in GenBank under the accession numbers KP189357 and KT962979, respectively.
